# Deep Learning-Based Prediction of Paresthesia after Third Molar Extraction: A Preliminary Study

**DOI:** 10.3390/diagnostics11091572

**Published:** 2021-08-30

**Authors:** Byung Su Kim, Han Gyeol Yeom, Jong Hyun Lee, Woo Sang Shin, Jong Pil Yun, Seung Hyun Jeong, Jae Hyun Kang, See Woon Kim, Bong Chul Kim

**Affiliations:** 1 Department of Oral and Maxillofacial Surgery, Daejeon Dental Hospital, Wonkwang University College of Dentistry, Daejeon 35233, Korea; kevin3001@naver.com (B.S.K.); wogus31001@gmail.com (J.H.K.); tologst@naver.com (S.W.K.); 2 Department of Oral and Maxillofacial Radiology, Daejeon Dental Hospital, Wonkwang University College of Dentistry, Daejeon 35233, Korea; hangyeol1214@gmail.com; 3 Advanced Mechatronics R&D Group, Korea Institute of Industrial Technology (KITECH), Gyeongsan 38408, Korea; ljh0325@kitech.re.kr (J.H.L.); welldone@kitech.re.kr (W.S.S.); rebirth@kitech.re.kr (J.P.Y.); shjeong@kitech.re.kr (S.H.J.)

**Keywords:** machine learning, third molar, tooth extractions, machine intelligence, inferior alveolar nerve injuries

## Abstract

The purpose of this study was to determine whether convolutional neural networks (CNNs) can predict paresthesia of the inferior alveolar nerve using panoramic radiographic images before extraction of the mandibular third molar. The dataset consisted of a total of 300 preoperative panoramic radiographic images of patients who had planned mandibular third molar extraction. A total of 100 images taken of patients who had paresthesia after tooth extraction were classified as Group 1, and 200 images taken of patients without paresthesia were classified as Group 2. The dataset was randomly divided into a training and validation set (n = 150 [50%]), and a test set (n = 150 [50%]). CNNs of SSD300 and ResNet-18 were used for deep learning. The average accuracy, sensitivity, specificity, and area under the curve were 0.827, 0.84, 0.82, and 0.917, respectively. This study revealed that CNNs can assist in the prediction of paresthesia of the inferior alveolar nerve after third molar extraction using panoramic radiographic images.

## 1. Introduction

Deep learning has developed rapidly in recent years, making it possible to automatically extract information in the medical field, from diagnosis using medical imaging to analysis of activity and emotional patterns [1,2,3]. The deep convolutional neural network (CNN), a type of deep learning, has been widely applied to medical images due to its high performance in detection, classification, quantification, and segmentation [4,5,6,7,8]. It can reduce the labor of experts and detect image information that may be missed by humans.

In the field of dentistry, research has been conducted about the detection and classification of anatomical variables [9,10], periapical lesions [11,12], dental caries [13,14,15], periodontitis [16], and benign tumors and cysts [17]. Deep learning analysis has also been applied to cephalometric images, such as detection of landmarks [18], prediction of the necessity for orthognathic surgery [19], and detection of cranio-spinal differences [20]. In relation to extraction of the mandibular third molar, attempts have been made to segment the teeth and the inferior alveolar nerve (IAN) [21,22], and, recently, a study to determine the difficulty of extraction was also conducted [23].

Extraction of the mandibular third molar is one of the most common surgical procedures performed in oral and maxillofacial surgery. In addition to pain and swelling after its extraction, paresthesia of the inferior IAN is one of the most common complications to be considered. Past studies have reported that the incidence of IAN injury during extraction of the impacted third molar is 0.4–5.5%; although most such injuries recover spontaneously, less than 1% of cases may be permanent [24]. Changes of the sensation in the oral and facial area can interfere with speaking, chewing, and social interactions, which can lower the basic quality of the life [25]. The mechanism of neurosensory deficits by surgical extraction is complex, and treatment results are often disappointing [25]. Therefore, the prediction of the possibility of paresthesia of the IAN prior to extraction would be helpful in making a treatment plan, explaining the surgical procedure to the patient, obtaining consent for extraction, and performing the operation [26].

Predicting paresthesia before extraction with a panoramic radiographic image is difficult because complex factors can affect the process of extraction and each step is highly dependent on the experience of the surgeons. Nevertheless, the risk of this complication depends mainly on the position of the impacted tooth in relation to the IAN before extraction [27]. Therefore, we hypothesized that the possibility of paresthesia after mandibular third molar extraction may be predicted through various information observed in the panoramic images using deep learning algorithms.

## 2. Materials and Methods

### 2.1. Datasets

A total of 300 panoramic radiographs of patients who underwent mandibular third molar extraction at the Department of Oral and Maxillofacial Surgery of the Wonkwang University Daejeon Dental Hospital from 2015 to 2019 were selected retrospectively (mean age: 32.0 years, age range: 17 to 62 years, 168 males and 132 females).

Only patients who had an appropriate neurosensory examination after extraction were included. Exclusion criteria were patients who were incapable of adequate neurosensory examination, with facial trauma, history of neurological disorders, vascular diseases, severe periodontal disease, or any other intraosseous disease which could affect the extraction. Patients who took psychotropic drugs or had undergone orthognathic surgery were also excluded. We used our own custom annotation tool for labeling.

Three experienced oral and maxillofacial surgeons performed extraction with the same operating technique, and the patients were monitored after the extraction process in the same way. To assess the paresthesia of IAN, all patients were clinically evaluated for paresthesia using a two point discrimination test seven days after extraction. In this test, while the patients closed their eyes, two tips of a non-sharp caliper touched the skin with light pressure. It was checked whether one point was recognized, and the distance was increased by 1 mm until the distance was recognized by two points [27]. Distances 15 mm greater than the preoperative values were considered abnormal. The period of paresthesia was not taken into account; we only investigated whether or not it occurred.

The panoramic radiographs of the patients were obtained using a PCH-2500^®^ (Vatech, Hwaseong, Korea) or ProMax^®^ (Planmeca, Helsinki, Finland) according to user’s manuals (72 kVp, 10 Ma 13.5 for Vatech; 72 kV, 12 mA, 15.8 for Planmeca).

A total of 100 images of patients with paresthesia after extraction were assigned to Group 1. A total of 200 images of patients without abnormal sensation were classified as Group 2. There was no discrepancy or selection bias between Group 1 and Group 2. This classification was representative of these groups in the general population [24,25,26,27].

This study was performed in accordance with the guidelines of the World Medical Association Helsinki Declaration for biomedical research involving human subjects and was approved by the Institutional Review Board of Daejeon Dental Hospital, Wonkwang University (W2003/002-001). Written or verbal informed consent was not obtained from any participants because the IRB waived the need for individual informed consent as this study applied a non-interventional retrospective design and all data were analyzed anonymously.

### 2.2. Preprocessing and Image Augmentation

The original images were taken using various panoramic imaging devices with various fields of view; hence, their sizes were 3324 × 1672 pixels, 2872 × 1504 pixels, and 2832 × 1376 pixels. The images were all resized to 2400 × 1200 pixels. Histogram equalization was used to reduce the difference in brightness and contrast between images. Image augmentation reduces overfitting and improves generalization performance. Rotation by an angle of ±10°, shifting in the range of 10%, and a horizontal flip were used.

The region of interest (ROI) was set manually to define the bounding box in the mandibular third molar area with the consensus of one oral and maxillofacial radiologist and one oral and maxillofacial surgeon. This included the crown and root of the mandibular third molar, and the shape was rectangular.

The process of finding the location of the mandibular third molar and setting it as the ROI was defined as the detection process, and the process of evaluating whether paresthesia occurred after extraction was defined as the classification process. The panoramic radiographic image contains information of the left and right sides, and, therefore, we divided an image into half and counted the ROIs again.

The dataset was randomly divided into a training and validation set (n = 150 [50%]), and a test set (n = 150 [50%]). CNNs of SSD300 and ResNet-18 were used for machine learning, and the detection process was trained.

### 2.3. Architecture of the Deep Convolutional Neural Network and Training Details

**Figure 1:**
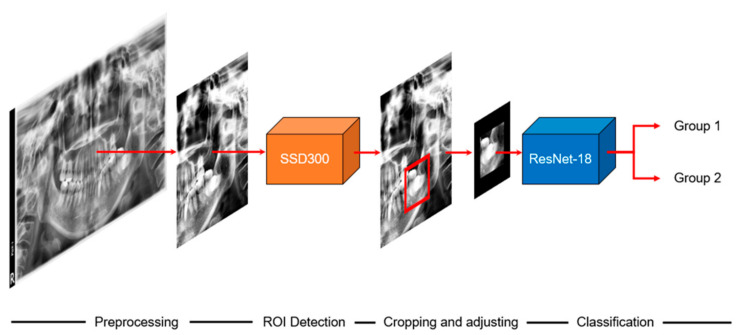
Overall scheme of this study. ROI: region of interest. Group 1: paresthesia after mandibular third extraction. Group 2: without paresthesia.

**Figure 2:**
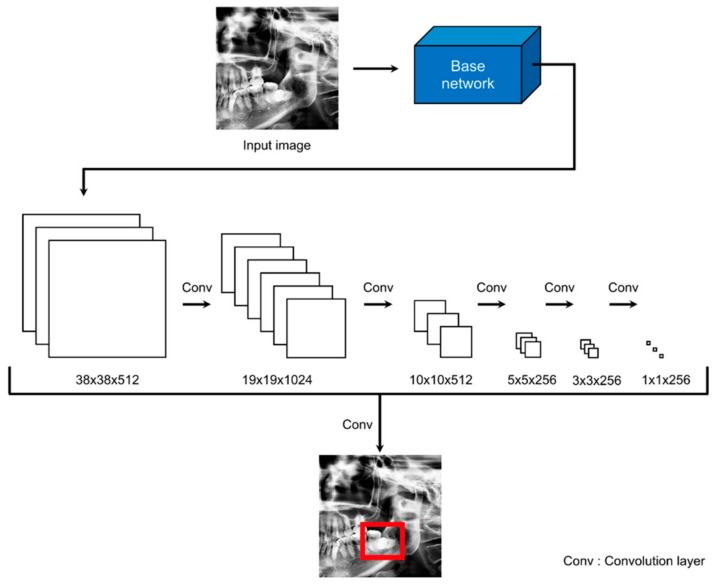
SSD300 architecture. The red box means ROI.

**Figure 3:**
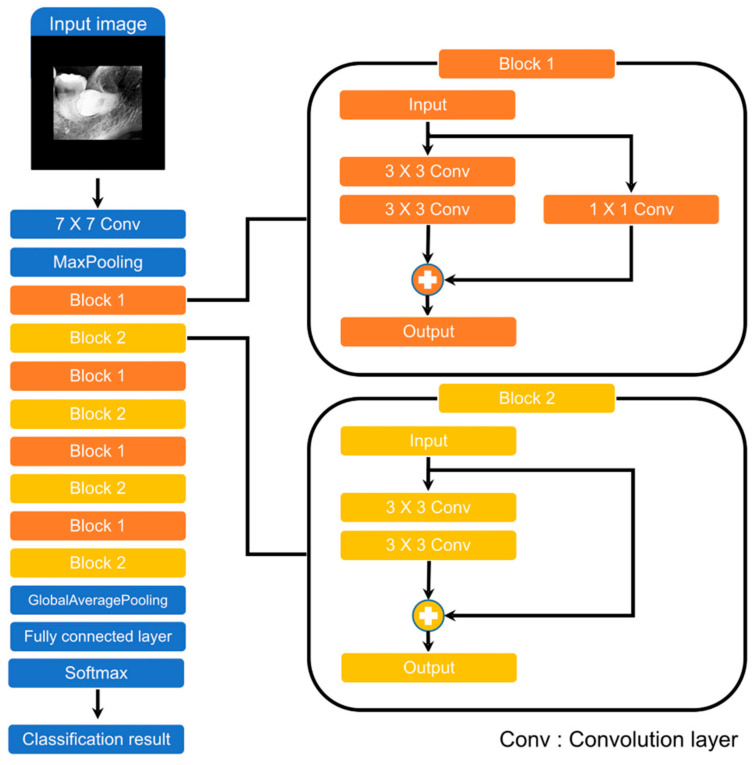
ResNet-18 architecture.

The overall scheme of this study is illustrated in Figure 1. When we input a panoramic image, the detection model sets the ROI to focus, and the classification model predicts paresthesia. The CNNs used in the detection and classification models of this study are shown in Figure 2 and Figure 3, respectively. The classification task is to assign the class label of the input example. The CNNs for classification consist of a feature extraction module and a classifier module. The feature extraction module extracts the features of an image using the convolution filter included in the convolutional layer. The classifier module determines the category of the input image using the extracted features. The object detection task not only recognizes the object, but also estimates the location of the object. The CNNs for object detection consist of a feature extraction module and a detection module. The detection module estimates the location of the object using the extracted features. SSD300 was used for detection model training, and ResNet-18 was used for classification model training.

### 2.4. Model for ROI Detection

We added a detection module for the ROI setting using SSD300. The Single Shot MultiBox Detector (SSD) [28] is a representative network of one-stage detectors that performs localization to locate objects and solves classification problems to recognize objects. One-stage detectors are easier to integrate into systems because of their simpler architecture than two-stage detectors. The architecture of SSD300 is illustrated in Figure 2. The SSD uses VGG16 [29] as the base network and creates multi-scale feature maps using extra feature layers from feature maps extracted from the base network. Thus, the performance of an SSD is improved because of its ability to predict various scales. The input size of SSD300 was 300 × 300 pixels. The preprocessed images were resized from 1200 × 1200 pixels to 300 × 300 pixels and used as the input. The coordinates of the detected bounding box were the result of using 300 × 300 pixels, and, hence, we rescaled the bounding box.

### 2.5. Classification Model

We performed prediction of paresthesia using the ROI as the input to the classification model. The ROIs obtained through the detection model had different sizes. However, for efficient training, we created a batch by making the images the same size. If the extracted ROIs are resized to the same width and height, the proportion and shape of the objects in the image are deformed. The objects in the ROIs of this study were the crown and root of the mandibular third molar. To avoid the deformation of these objects, we added zero values around the ROIs to make them the same size of 600 × 600 pixels.

We used ResNet as the classification model [30]. ResNet, which is also a CNN, has demonstrated excellent image recognition performance. ResNet-18 was chosen from a variety of ResNet models. It was chosen due to its relatively low number of parameters to reduce overfitting due to small datasets. Figure 3 illustrates the architecture of ResNet-18. It consists of a 7 × 7 convolution layer, 2 pooling layers, 8 blocks, and a fully connected layer. The blocks are the most important elements of ResNet and are called residual blocks. These blocks use shortcut connections. Shortcut connections help reduce the vanishing and exploding gradient problems when optimizing parameters using backpropagation. The difference between Block 1 and Block 2 is the presence or absence of a 1 × 1 convolution layer. This is used to ensure the same dimension when the input and output dimensions are different.

### 2.6. Training Details

**Figure 4:**
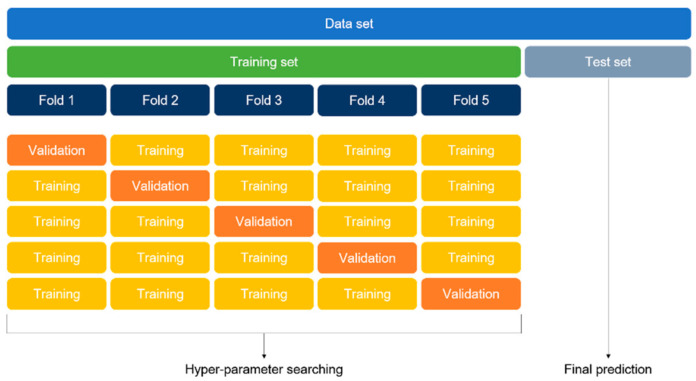
Five-fold cross-validation method.

The optimal hyper-parameters were found using the K-fold cross-validation method. The K-fold cross-validation method divides the entire training set into K folds and uses one of the folds for validation and the remainder of the folds for training. The average of accuracy from all the folds is the overall validation accuracy. We repeated the cross-validation method to identify the optimal hyper-parameter and then trained on the entire training set. The final accuracy was the prediction result on the test set. This approach is computationally expensive but does not waste data for validation. In this study, the 5-fold cross-validation method was applied. Figure 4 illustrates the 5-fold cross-validation method, which was equally applied to both ROI detection and classification models.

The classification model (Resnet-18) and the backbone network of the SSD (VGG16) were initialized using ImageNet pre-trained weights. The classification model was trained 10 times using the optimal hyper-parameters and training datasets. We used stochastic gradient descent with a momentum of 0.9 and batch size of 2. The learning rate was initially 0.001 and then 0.0001 after 80 epochs. The number of epochs was fixed at 100. The ROI detection model was trained 1 time using the optimal hyper-parameters and training datasets. We used stochastic gradient descent with a momentum of 0.9 and batch size of 16. The learning rate was initially 0.001 and then 0.0001 after 40 epochs. The number of epochs was fixed at 50. Because there was no validation set, we used the final result of training without early stopping. In addition, the Keras API in Tensorflow was used for training and testing.

The hardware and software environment specifications are as follows.

- CPU: Intel i7-7700K
- RAM: 32 GB
- GPU: NVIDA Geforce RTX 2080 Ti
- Framework: Tensorflow 2.1.0
- OS: Windows 10

### 2.7. Statistical Analysis

**Figure 5:**
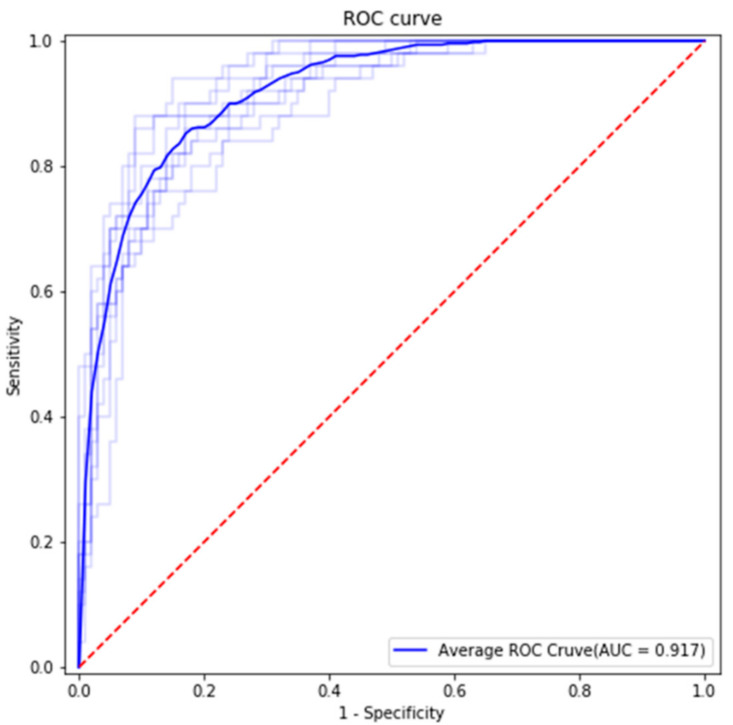
Classification results. The ROC curve was created by plotting sensitivity against (1-specificity) at various threshold settings. AUC is area under the curve. The blue line is the average ROC curve. The light blue lines are the ROC curves of each classification model.

**Figure 6:**
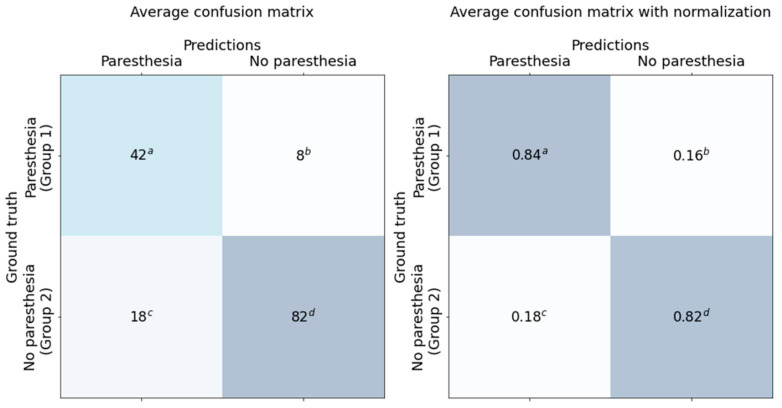
Classification results—confusion matrix. Prediction: Expected paresthesia to appear after extraction as a result of deep learning. Group 1: Paresthesia of inferior alveolar nerve actually appeared after mandibular third extraction. Group 2: No paresthesia of inferior alveolar nerve actually appeared after mandibular third extraction. *^a^* True positive. *^b^* False negative. *^c^* False positive. *^d^* True negative.

Statistical analysis was performed using accuracy, sensitivity, specificity, and area under the curve (AUC) calculated based on the confusion matrix and receiver operating characteristic (ROC) curve, as shown in Figure 5 and Figure 6. The AUC is shown in Figure 5.

## 3. Results

The classification results for the dataset are shown in the confusion matrix (Figure 6) and ROC curve (Figure 5). All results are the average of 10 models trained with the same training details and training dataset.

We used 150 panoramic images as test data. The test dataset included 50 cases of Group 1 and 100 cases of Group 2. The average accuracy, sensitivity, specificity, and AUC were 0.827, 0.84, 0.82, and 0.917, respectively. Average false positive rate (FPR), false negative rate (FNR), positive prediction value (PPV), and negative prediction value (NPV) were 0.098, 0.3, 0.84, and 0.82, respectively.

**Figure 7:**
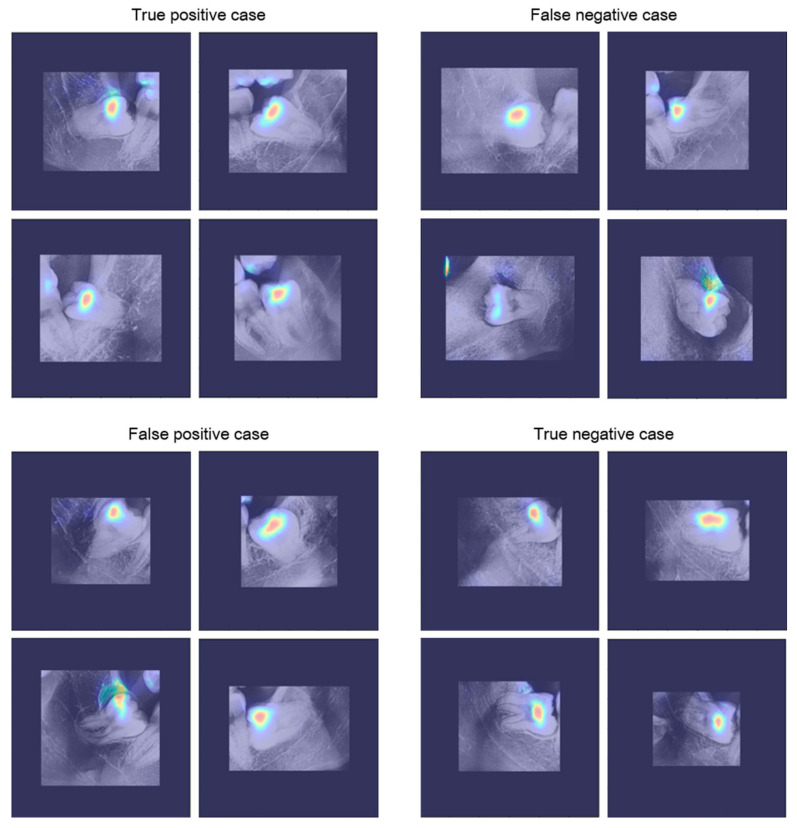
CNN visualization results.

Figure 7 shows the results of applying the visualization method to the trained CNN. CNN visualization methods are used to interpret the predictions of the network and provide a basis for judgment. In this study, the VisualBackProp [31] method was applied as a visualization method. The degree of judgment contribution is expressed as a color from blue (low) to red (high). Observing the visualization results, it can be seen that the trained CNN makes decisions based on the information around the third molars.

## 4. Discussion

In this study, we used a two-step process to predict paresthesia before operation. The first step was to identify each ROI using the detection model. Because ROI detection is based on the context feature, the sizes of the images were reduced to 300 × 300 pixels and used as the input to the model. The second step was prediction using the classification model and the detected ROIs. The ROIs were cropped from the original images while maintaining their resolution to avoid loss of fine feature information.

There are two advantages to using a two-step process. First, it is possible to focus on meaningful information by removing unnecessary information. The panoramic images contain a large volume of information in addition to that needed for prediction. Classification performance was improved by setting the ROIs using the detection model and focusing on meaningful information. Second, the size of the input image is reduced. In general, machine learning requires more data as the dimension of the data increases. Therefore, by using an image smaller than the original, we were able to train the model sufficiently with a smaller quantity of data. This also significantly reduces computational costs compared to using the original image.

In recent years, successful applications of CNNs in various fields have been reported. In the field of medical imaging, CNN architectures have shown efficient results similar to or better than those of human experts [1,2,3,4,5,6,7,8]. In this study, we proved that a CNN architecture can accurately detect mandibular third molars and predict paresthesia before third molar extraction.

Paresthesia of IAN can be predicted, thus confirming the anatomical relationship between the nerve canal and dental roots. This is possible using panoramic radiographic images. In particular, narrowing/diversion of the mandibular canal, interruption of the white line of the canal, and deflection/darkening of the roots are the main indicators. The depth and type of impaction of the mandibular third molar also affects paresthesia. Mesioangular and deeper horizontal impactions are known to increase the likelihood of paresthesia. In this study, it was found that CNNs can intuitively predict the paresthesia of IAN by synthesizing several factors, including these indicators.

It is clear that numerous other factors also affect the paresthesia of IAN. These include IAN exposure during extraction, anesthetic techniques, the experience of the surgeon, and the surgical technique. The main limitation of this study is that it was not able to consider the combination of these factors because the current level of CNNs cannot easily reflect these factors objectively. Nevertheless, the evaluation of the accuracy, sensitivity, specificity, and AUC at this level with panoramic images alone was found to be clinically acceptable, although not excellent [24,25,26,27]. If a three-dimensional version of this study using computed tomography is conducted in the future, it will be possible to reduce the number of false positives and false negatives.

## 5. Conclusions

Various factors, such as the operating technique, affect paresthesia after third molar extraction. In addition, the stereoscopic anatomical relationship between IAN and the third molar cannot be determined from a two-dimensional panoramic image alone. As a result, this study found approximately 18% false positives and 16% false negatives. Therefore, the results of this study cannot be absolute criteria for the prediction of paresthesia after third molar extraction. However, this study, which revealed that CNNs can assist in the prediction of paresthesia after third molar extraction, will be the basis for future research.

## Data Availability

The datasets generated during and/or analyzed during the current study are available from the corresponding author on reasonable request but is subject to the permission of the Institutional Review Boards of the participating institutions.
